# CTSG Suppresses Colorectal Cancer Progression through Negative Regulation of Akt/mTOR/Bcl2 Signaling Pathway

**DOI:** 10.7150/ijbs.82000

**Published:** 2023-04-17

**Authors:** Shixin Chan, Xu Wang, Zhenglin Wang, Youwen Du, Xiaomin Zuo, Jiajie Chen, Rui Sun, Qing Zhang, Li Lin, Yang Yang, Zhen Yu, Hu Zhao, Huabing Zhang, Wei Chen

**Affiliations:** 1 Department of General Surgery, The First Affiliated Hospital of Anhui Medical University, Hefei 230032, Anhui, China.; 2 Department of Biochemistry and Molecular Biology, Metabolic Disease Research Center, School of Basic Medicine, Anhui Medical University, Hefei 230032, Anhui, China.; 3 Affiliated Chuzhou Hospital of Anhui Medical University, First People's Hospital of Chuzhou, Chuzhou 239000, Anhui, China.; 4 School of Life Sciences, Anhui Medical University, Hefei 230022, Anhui, China.; 5 Department of Dermatology, First Affiliated Hospital of Anhui Medical University, Hefei 230022, Anhui, China.; 6 Anhui Provincial Institute of Translational Medicine, Hefei 230022, Anhui, China.

**Keywords:** CTSG, colorectal cancer, Akt, mTOR, Bcl2

## Abstract

Colorectal cancer (CRC) is the most common gastrointestinal tumor worldwide, which is a severe malignant disease that threatens mankind. Cathepsin G (CTSG) has been reported to be associated with tumorigenesis, whereas its role in CRC is still unclear. This investigation aims to determine the function of CTSG in CRC. Our results indicated that CTSG was inhibited in CRC tissues, and patients with CTSG low expression have poor overall survival. Functional experiments revealed that CTSG overexpression suppressed CRC cell progression *in vitro* and *in vivo,* whereas CTSG suppression supports CRC development cells* in vitro* and* in vivo*. Mechanistically, CTSG overexpression suppressed Akt/mTOR signaling mechanism and elevated apoptotic-associated markers, and CTSG silencing activated Akt/mTOR signaling mechanisms and inhibited apoptotic-associated markers. Furthermore, the Akt suppression signaling pathway by MK2206 abolishes CTSG-silenced expression-induced cell viability and Bcl2 up-regulation *in vitro* and* in vivo*. Altogether, these outcomes demonstrate that CTSG may act as a tumor suppressor gene *via* Akt/mTOR/Bcl2-mediated anti-apoptotic signaling inactivation, and CTSG represents a potential therapeutic target in CRC.

## Introduction

Colorectal cancer (CRC) is the most prevalent gastrointestinal cancer globally 1, which is a malignant disease with high mortality; the number of individuals with CRC worldwide is expected to increase to 2.2 million and nearly 1.1 million deaths by 2030 2. Evidence-based medicine has shown that the occurrence of CRC is closely related to gene mutations, family history, high-fat diet, inflammation, immunity, and intestinal microbes 3. Complex signaling pathways, including Wnt, Jak/Stat, PI3K/Akt, Mapk, TGF-β, NF-κB, and Notch, have a significant role in CRC pathogenesis 4-10. Although with the improvement of surgical techniques, chemotherapy, targeted therapy, immunotherapy, and other treatment measures, the curative effect of CRC has been unsatisfactory in the past decade 11, 12. Therefore, it is important to explore novel pathways that regulate CRC occurrence and progression to provide novel therapeutic targets.

The protein kinase B/mammalian target of rapamycin (Akt/mTOR), as a crucial cancer-related signaling mechanism, its dysregulation or activation has been reported to be involved in tumorigenesis and tumor cellular functions, like growth, apoptosis, migration, and invasion 13, 14. The inhibitors or drugs targeting the Akt/mTOR signaling pathway have been clinically studied 15, 16. B-cell lymphoma 2 (Bcl2), an important cancer-associated regulator, plays a crucial role in cell apoptosis 17, and its presence or absence determines the susceptibility of cancer cells to chemotherapy 18. Previous studies have suggested that Bcl2 may be Akt signaling pathway downstream, and the Bcl2 expression level is consistent with the Akt phosphorylation level 19, 20.

Cathepsins, which belong to the serine, aspartic protease, or cysteine classes, are ubiquitously expressed in human tissues and perform different biological functions 21. Increasing evidence suggests that the Cathepsin family is associated with CRC occurrence and progression. An Investigation indicated that miR-106b-5p might inhibit CRC cell metastasis and invasion by targeting Cathepsin A (CTSA) 22. Bian et al. 23 revealed that CTSB induced CRC metastasis and invasion by down-regulating the p27 expression. Li et al. 24 identified that CTSK secretion stimulated by intestinal microorganisms mediated TLR4-dependent M2 macrophage polarization and promoted metastasis in CRC. Gormley et al. 25 showed that CTSS could be a diagnostic marker for CRC and may increase 5-Fu and folic acid therapeutic sensitivity. Many studies have indicated that CTSG, a member of the Cathepsin family, functions as a cancer suppressor in many tumors, inhibiting the malignant features and cancers development, like breast, bladder, oral squamous cell cancer, and acute myeloid leukemia 26-29. However, CTSG role in CRC oncogenesis has rarely been studied.

Herein, we evaluated the information published in The Cancer Genome Atlas (TCGA) database and performed quantitative PCR (qPCR), western-blot, and immunohistochemistry (IHC) staining; our results revealed that CTSG was substantially decreased in CRC tissues, and subjects with CTSG low-expression had poorer overall survival. CTSG expression correlated with tumor size, location, and TNM stage. Functional studies showed that CTSG overexpression suppressed CRC cell development* in vitro* and* in vivo*. Whereas CTSG suppression indicated the opposite phenomenon. Additionally, the Kyoto Encyclopedia of Genes and Genomes (KEGG) pathway analysis enriched Akt, mTOR signaling pathways, and apoptosis process. Moreover, Akt signaling pathway suppression by MK2206 abolishes CTSG-silenced expression-induced up-regulation of Bcl2 and cell viability *in vitro* and *in vivo*. Our data revealed that CTSG could be a critical tumor suppressor in CRC.

## Materials and Methods

### Patients and tissue specimens

Twenty CRC specimens and corresponding nearby normal tissues were chosen for CTSG mRNA and protein level analysis by qPCR and western blot. A total of 112 CRC specimens and 41 randomly selected normal tissues from January 2014 to December 2016 were chosen for IHC assays. All tissue specimens were obtained from the First Affiliated Hospital of Anhui Medical University (Hefei, China) with the patients' agreement.

### Bioinformatics analyses and clinical data

The data from TCGA database was employed to compare CTSG mRNA expression levels in normal and CRC tissues through xiantao website (https://www.xiantao.love/). **Table S1** illustrates the relationships between CTSG expression and clinicopathological characteristics. Pie charts were used to demonstrate the percentage of clinicopathological features in high- and low-risk groups employing the Chi-squared test. The KM curve was created to compare the patients' overall survival (OS) in two groups. To investigate if CTSG expression was an independent predictive factor for patients with CRC, Uni- and multivariate Cox regression analyses were conducted. A nomogram model may predict patient prognosis more correctly based on the Cox regression analysis findings. To demonstrate the effectiveness of the nomogram model, CTSG expression, and other clinicopathological characteristics, ROC analysis was created.

### Plasmids and reagents

The plasmids employed in this investigation are listed in **Table S2**. Annexin V-PE/7-AAD Apoptosis Kit was obtained from Vazyme BioTech Co. LTD. (Nanjing, China). MK2206 and SC79 were achieved from SparkJade (Shandong, China). Rabbit Two-step Kit used for immunohistochemistry staining was obtained from ZSJQ-BIO (Beijing, China). ZEN-BIO provided the goat anti-rabbit IgG (H&L) Alexa Fluor 594 utilized for immunofluorescence (Chengdu, China).

### Cell lines and cell culture

Human CRC cell lines (HT29, HCT116, SW480, and RKO), NCM460 (normal colonic epithelial cell), and HEK293T were acquired from the American Type Culture Collection (ATCC) and cultivated in Dulbecco's Modified Eagle Medium (DMEM, Gibco, USA), which was treated with 10% Fetal Bovine Serum (FBS, Lonsera, Australia) and 1% antibiotic agent (100 μg/mL streptomycin and 100 U/mL penicillin) at 37 °C in 5% CO_2_.

### Lentivirus packaging and construction of stable cell lines

Lentiviral packaging plasmids and plasmids carrying overexpression or knockdown target genes were added to 1.5 ml EP tubes in a particular proportion, and transfection reagent JetPRIME^®^ (Polyplus-transfection, Illkirch, France) was added and mixed with the plasmids. After incubation for 10 minutes (min) at room temperature, the plasmids were supplemented to HEK293T cells and cultured in the incubator. The virus supernatant was collected at 36 hours (h) and 72 h. The virus supernatant was collected after concentrated using PEG8000 overnight at 4℃, and then DMEM was added to obtain the virus. Lentiviral vectors were transfected into cells (HT29 and HCT116 for overexpression experiments, and HCT116 and RKO for knockdown experiments) before 5 µg/ml puromycin selection. qPCR and Western blots were used to confirm the stable CTSG overexpression and knockdown cells.

### Cell proliferation assay

MTT (Beyotime, China) and colony formation assay were employed to evaluate cell viability. Stably transfected cells were developed in 96-well plates at a density of 3 × 10^3^ per well for the MTT assay. Each well received 25 μl MTT for 1 h, followed by 100 μl DMSO for 10 min. The absorbance at 6 h (regarded as 0 h), 24 h, 48 h, 72 h, and 96 h were measured by a microplate analyzer at 490 nm. For the colony formation assay, stably transfected cells were grown in 6-well plates at 3 × 10^3^ per well. Colonies numbers were fixed with methanol and stained with 1% crystal violet after ten days of culturing.

### Flow cytometry assay

Cell apoptosis experiments were performed using flow cytometer. Briefly, the cells were collected following the apoptosis detection kit instructions when the cells reached 90% of the 6-well plates. After administering Annexin V-PE and 7-AAD for 10 min at room temperature in dark, the cell apoptosis rates were determined.

### Western blotting

Total proteins were extracted using RIPA buffer (Beyotime, China) treated with phosphatase and protease suppressor. Western blotting was conducted according to the previous protocol 30. The primary antibodies used in the study were anti-GAPDH, anti-Bax (Santa, USA), anti-Flag (Sigma-Aldrich, USA), anti-CTSG, anti-Bcl2 (Affinnity Biosciences, USA), anti-Akt/phospho-Akt, anti-mTOR/phospho-mTOR (Cell Signaling Technology, USA), cleaved-caspase3 (ABclonal, China), and anti-c-Myc (Proteintech, China).

### RNA extraction and qPCR

Total RNA was separated employing the TRIzol-based method (Invitrogen, USA), and RNA quality was determined by examining A260/A280 with Nanodrop^TM^ (Thermo Fisher Scientific Inc, USA). Utilizing a reverse transcription Kit (Thermo Fisher Scientific Inc, USA), 1 μg of total RNA was reverse transcribed into cDNA following the manufacturer's instructions. Using the iQ SYBR Green Supermix, the qPCR was conducted using the Roche LightCycler® 480II PCR equipment (Promega, USA). **Table S3** includes a list of the primer sequences employed in this investigation.

### RNA-sequencing and data analysis

Seqhealth Technology Co. LTD. (Wuhan, China). Briefly, total RNA was obtained from stable CTSG overexpression and control cells using TRIzol. The RNA integrity was confirmed using agarose gel electrophoresis. The Stranded mRNA Library Prep Kit for Illumina® (KC-Digital^TM^) (Seqhealth Technology Co. LTD., China) was used to create the RNA sequencing library, and the DNBSEQ-T7 sequencer was used to finalize the sequencing (MGI Tech Co. LTD., China). Raw sequencing data were used for standard RNA-seq analysis after the de-duplicated consensus sequence generation. The RNA-sequencing data have been deposited in NCBI under SRA accession numbers (SRA: PRJNA907192).

### Immunohistochemistry

CTSG protein expression levels in tissues were identified by IHC assay with a rabbit two-step Kit. After standard deparaffinization, rehydration, and antigen retrieval, the tissue portions were briefly treated with anti-CTSG antibody overnight at 4 °C. Then, they were incubated with HRP-conjugated secondary antibody, followed by visualization with DAB. Finally, hematoxylin was used to counterstain the specimens. CTSG protein expression levels were represented by the IHC score using ImageJ software described previously 31.

### Immunofluorescence

Tumor tissues from mice were embedded in paraffin after soaking in formalin. The tumor tissues were cut into 5 μm sections and incubated with the primary antibody Ki67 (ABclonal, Wuhan, China) at 4 °C overnight, following goat serum blocking for 30 min at 37 ℃. All samples were incubated with DAPI in dark for 10 min after being exposed to Goat anti-Ribbit IgG (H&L) Alexa Fluor 594 for 1 h. Finally, a fluorescent microscope was employed to examine the segments.

### Xenograft tumor growth model

All 5-week-old male BALB/c nude mice used in this investigation were reared in the SPF animal room following institutional ethics and standards (Animal Ethical Committee of Anhui Medical University, Hefei, China). The mice were provided by Gempharmatech Inc. in Nanjing, China. The subcutaneous tissue of the right flanks of mice was injected with stable CTSG overexpression or knockdown cells and control cells (3 × 10^6^ cells in 200 μl DMEM). Every two days up to the termination of the experiment, the weight of mice and the size of the tumors were measured. The formula used to determine the tumor volume was V = 1/2 × length × width × width. Finally, the tumors were obtained for pathological analysis after the mice were sacrificed.

## Results

### CTSG is down-regulated in human CRC tissues and colorectal cancer cell lines

**Figure 1:**
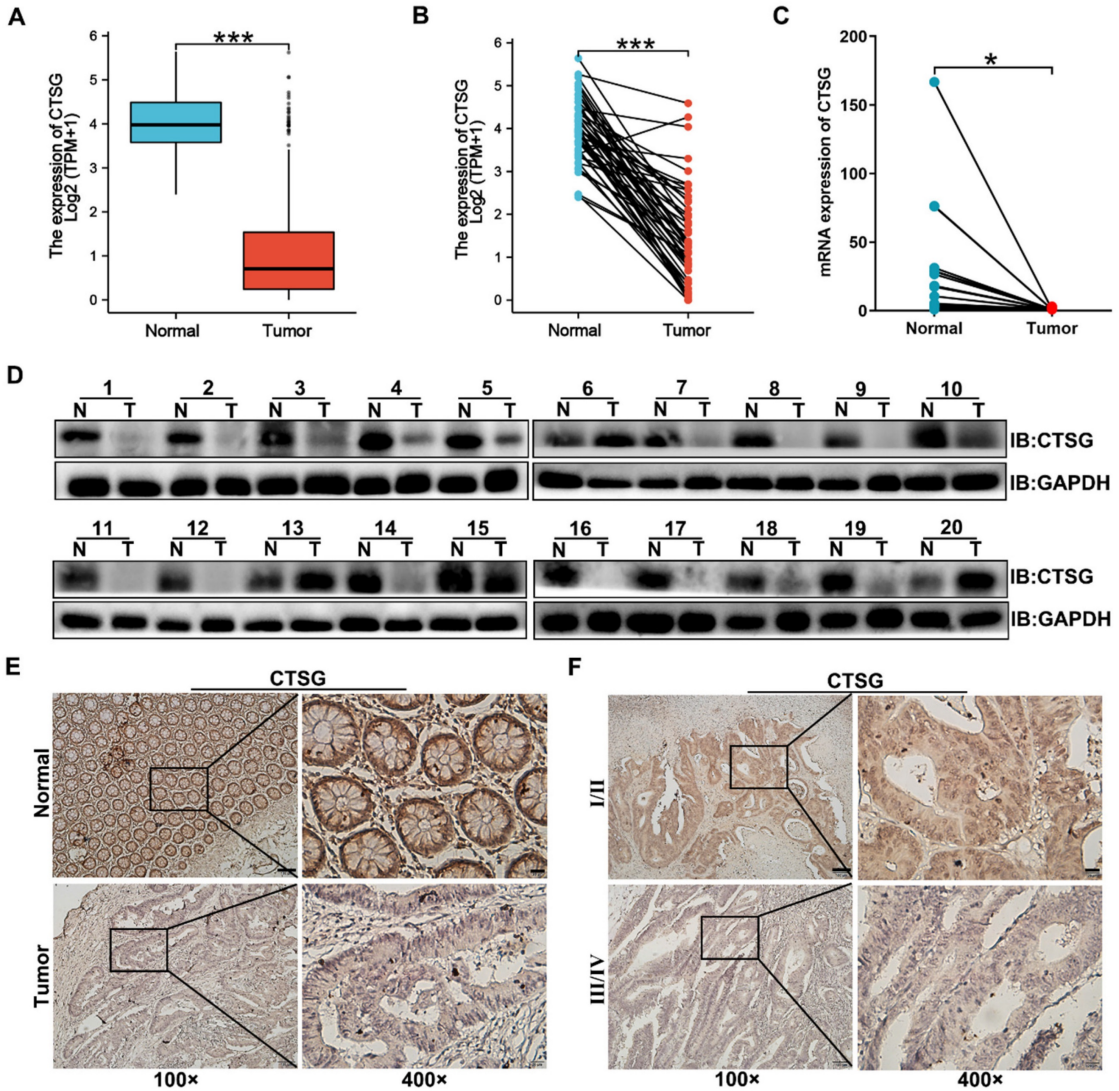
** CTSG expression is down-regulated in human CRC tissues.** (A**-**B) The data from TCGA showed that CTSG was significantly down-regulated in colorectal cancer tissues compared to the normal tissues (*P* < 0.001); (C) qPCR showed CTSG mRNA expression in 20 paired CRC and normal tissues (*P* < 0.05); (D) Western-blot analyzed the CTSG protein expression levels in 20 paired CRC and normal tissues; (E) Representative IHC staining of CTSG expression in CRC tissues and normal tissues; (F) Representative IHC staining of CTSG expression in TNM stage Ⅰ/Ⅱ and TNM stage Ⅲ/Ⅳ. Scale bar: 100 μm (100 ×) or 20 μm (400 ×). *P < 0.05, ***P < 0.001.

Previous studies reported that CTSG is associated with carcinogenesis, but its function in CRC development remains unclear. To investigate CTSG's role in colorectal cancer, we compared gene expression of CTSG in normal and primary tumor tissues using TCGA. The outcomes revealed that CTSG was significantly inhibited in CRC tissues compared to the adjacent cancerous tissues (**Figures 1A-B**). qPCR and Western-blot results identified that CTSG mRNA (**Figure 1C**) and protein levels (**Figure 1D**) were down-regulated in 20 paired CRC compared to healthy tissues. Furthermore, we detected the CTSG protein level in 112 CRC tissues, and 41 randomly selected normal tissues using immunohistochemistry (IHC). The IHC score for each sample is shown in **Figure S1**. The CTSG expression is elevated in cancer tissues than in adjacent cancerous tissues (**Figure 1E**). The rate of high CTSG expression was substantially greater in adjacent cancerous tissues than in CRC tissues [28/41 (68.3%) vs. 42/112 (37.5%), *P* < 0.001] (**Table S4**). Moreover, CTSG expression in TNM stage Ⅰ/Ⅱ was more than in TNM stage Ⅲ/Ⅳ (**Figure 1F**). These data suggest that CTSG deficiency may be related to the growth of CRC.

### Low CTSG expression predicted a poor prognosis in CRC patients

**Figure 2:**
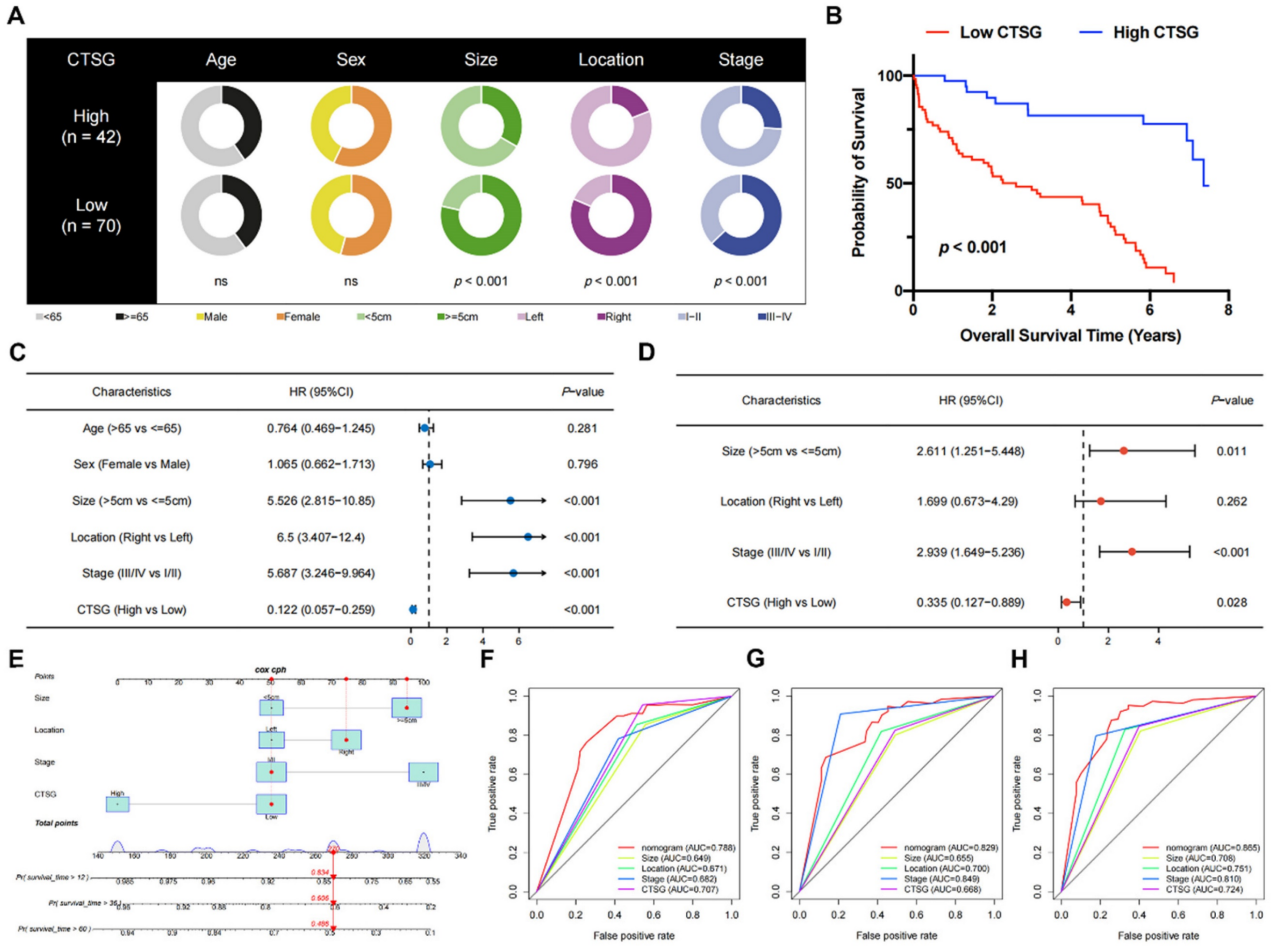
** The correlations between clinicopathological features and CTSG expression.** (A) Pie charts showing the Chi-squared test of clinicopathologic factors in high- and low-CTSG groups; (B) Patients in the high-CTSG group experienced a longer survival time tested by the Kaplan-Meier (KM) method; (C**-**D) Forest plots of univariate (C) and multivariate (D) Cox regression analyses in patients with CRC; (E) Nomogram model construction using CTSG and other clinical features; (F**-**H) 1- (F), 3- (G), and 5-year (H) AUC of the nomogram model, CTSG expression, and other clinical-pathological features were presented.

Pie charts suggested that individuals with low CTSG were more susceptible to CRC with greater tumor sizes (*P* < 0.001), right locations (*P* < 0.001), and more progressive pathological phase (*P* < 0.001) than individuals with high CTSG (**Figure 2A**). Additionally, the KM curve showed that patients with low CTSG had a considerably poorer prognosis than those with high CTSG (P < 0.001) **(Figure 2B)**. The multivariate analysis **(Figure 2D)** accounted for factors having a P-value <0.05 in the univariate analysis **(Figure 2C)**. After multivariate analysis, CTSG expression, phase, site, and size of the tumor all remained significant, and these four variables were included in the nomogram model **(Figure 2E)**. **Figures 2F-H** also had the nomogram model AUC, CTSG expression, and other clinical-pathological characteristics. The nomogram AUC was 0.788, 0.829, and 0.865 for the 1-, 3-, and 5-year survival, respectively.

### Overexpression of CTSG inhibits proliferation and induces apoptosis of CRC cells in *vitro*

**Figure 3:**
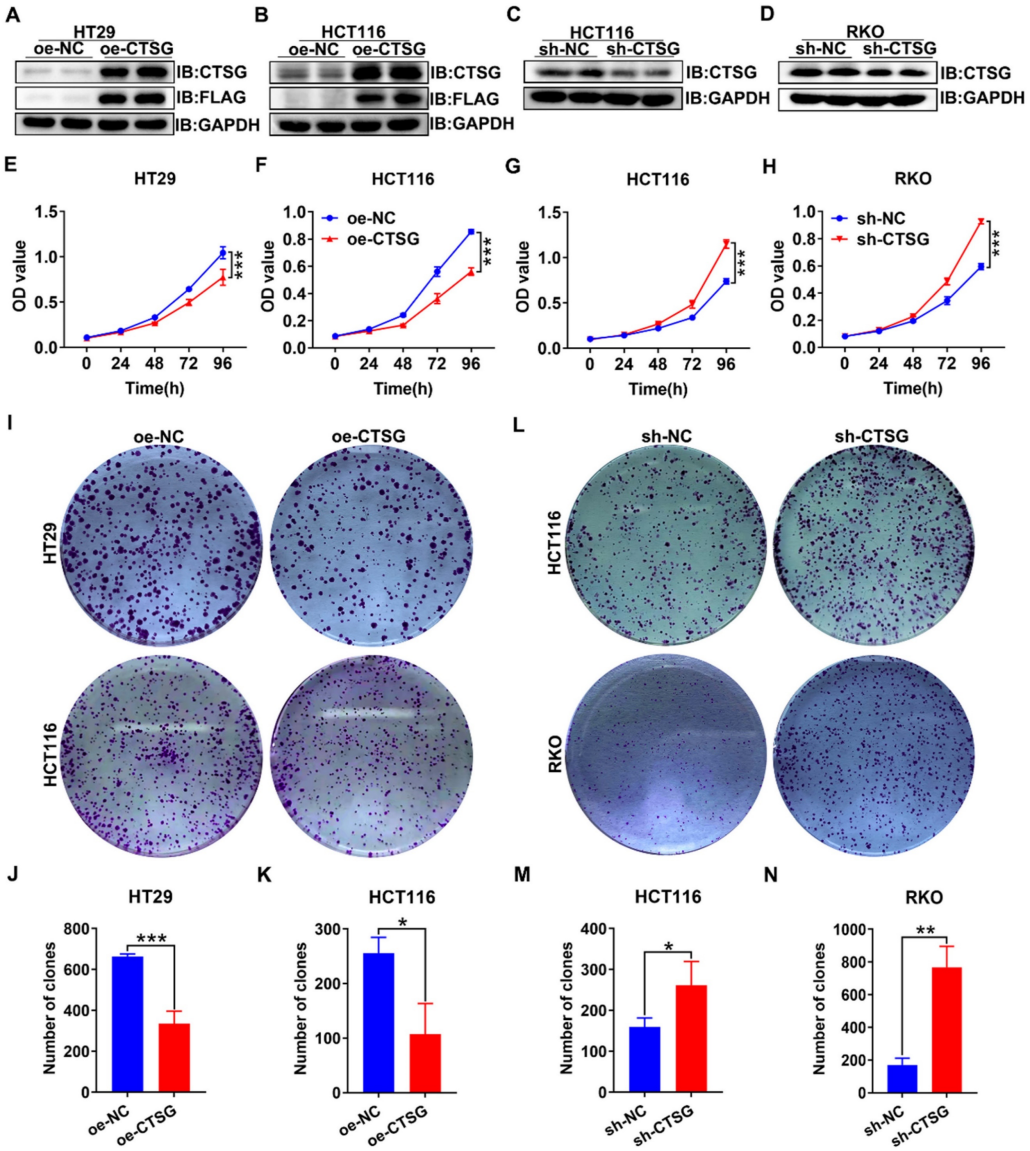
** CTSG inhibits the proliferation of CRC cells *in vitro*.** (A**-**D) Overexpression efficiency of HT29 (A) and HCT116 (B) cell lines, and knockdown efficiency in HCT116 (C) and RKO (D) cell lines; (E**-**F) Effects of CTSG overexpression on the proliferation of HT29 (E) and HCT116 (F) cells were monitored by MTT assays; (G**-**H) Effects of CTSG down-regulated expression on the proliferation of HCT116 (G) and RKO (H) cells were monitored by MTT assays; (I) Effects of CTSG overexpression in HT29 and HCT116 cells on colony formation; (J**-**K) Statistical results showed that the number of clones indicated in I; (L) Effects of CTSG down-regulated expression in HCT116 and RKO cells; (M**-**N) Statistical results showed the number of clones indicated in L. *P < 0.05, **P < 0.01, ***P < 0.001.

As unlimited growth is a cancer hallmark 32, we next investigate the role of CTSG in developing CRC. Firstly, we found the endogenous CTSG expression was greater in several CRC cell lines than in NCM460 (**Figures S2A-B**). Based on the above results, we constructed CTSG stable overexpression HT29 and HCT116 cell lines and CTSG stable knockdown expression HCT116 and RKO cell lines. The overexpression and knockdown efficiency were shown in **Figures 3A-D**. MTT assays indicated that upregulation of CTSG inhibited CRC proliferation compared with control groups (**Figures 3E-F**), whereas CTSG knockdown enhanced CRC growth (**Figures 3G-H**). Furthermore, colony formation assays showed that CTSG-enforced expression decreased colonies amount (**Figures 3I-K**), and its silencing expression showed contrary results (**Figures 3L-M**). Though, CTSG upregulation had no substantial impact on CRC invasion and metastasis. These results suggested that CTSG inhibits CRC cell proliferation *in vitro*.

**Figure 4:**
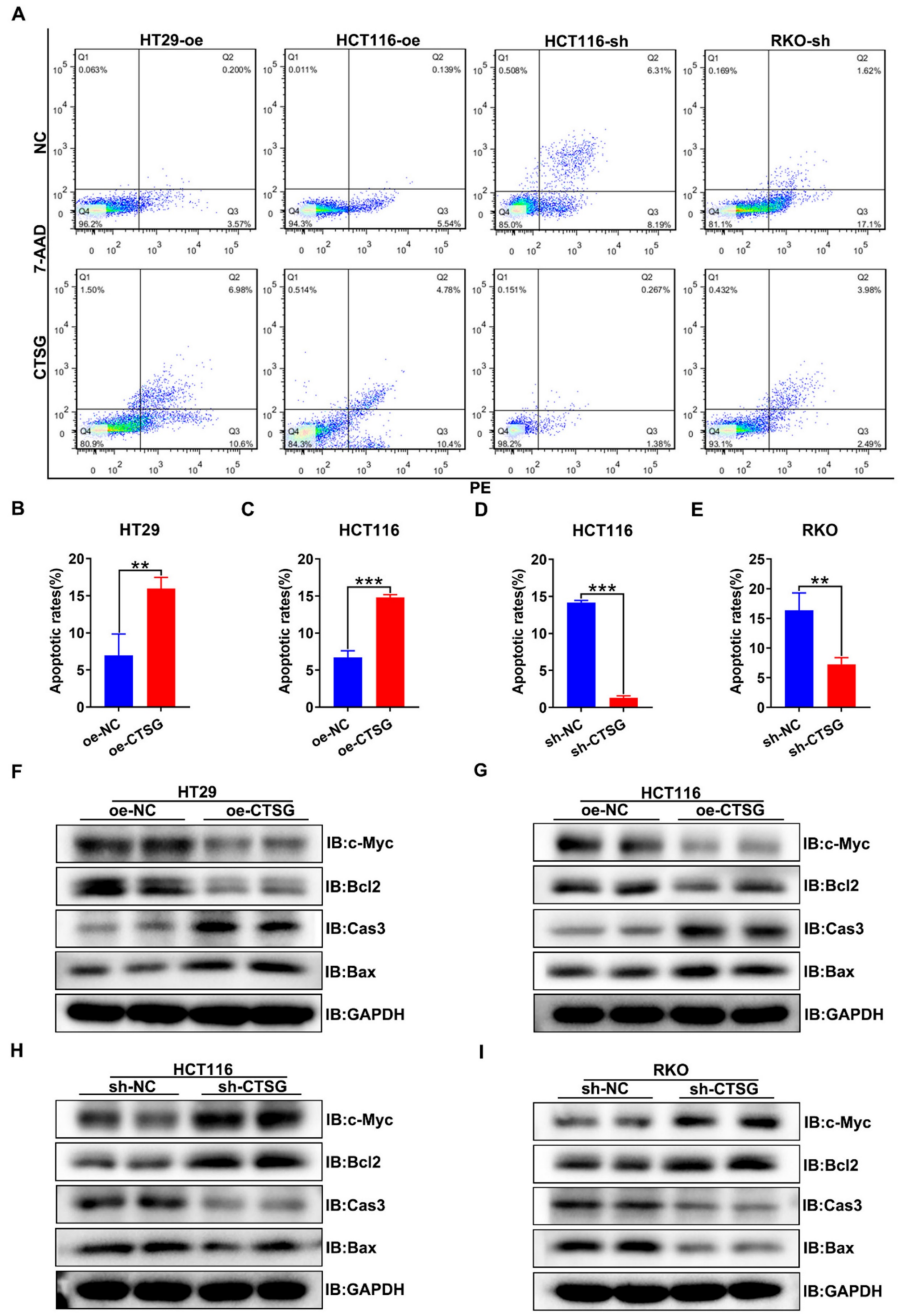
** CTSG induces apoptosis of CRC cells *in vitro*.** (A) Up-regulation or down-regulation of CTSG expression affected the apoptosis of colorectal cancer cells; (B**-**E) Statistical analysis indicated the difference in apoptosis rates in CRC cells, up-regulation of CTSG expression could promote CRC cell apoptosis (4B-C), whereas down-regulation of CTSG expression restrains CRC cell apoptosis (4D-E); (F**-**I) Apoptosis-related protein expression was examined by western-blot with CTSG overexpression in HT29 (F) and HCT116 (G) or with CTSG down-regulated expression in HCT116 (H) and RKO (I). **P < 0.01, ***P < 0.001.

Furthermore, we discover whether CTSG influences the apoptosis of CRC cells *in vitro*. It was found that CTSG expression up-regulation might promote the apoptosis of CRC cells compared with control groups (**Figures 4A-C**), whereas CTSG expression down-regulation reduces CRC cells apoptosis (**Figures 4A** and** D-E**). Western-blot analysis revealed that CTSG overexpression might inhibit the anti-apoptotic protein Bcl2 and proliferative protein c-Myc expression levels and promote the apoptotic proteins Caspase3 and Bax expression (**Figures 4F-G** and **Figures S3A-B**). In contrast, CTSG silencing expression might increase the Bcl2 and c-Myc protein expression levels and decrease the Caspase3 and Bax protein expression (**Figures 4H-I** and **Figures S3C-D**).

### CTSG mediates Akt/mTOR signaling pathway in CRC cell lines

**Figure 5:**
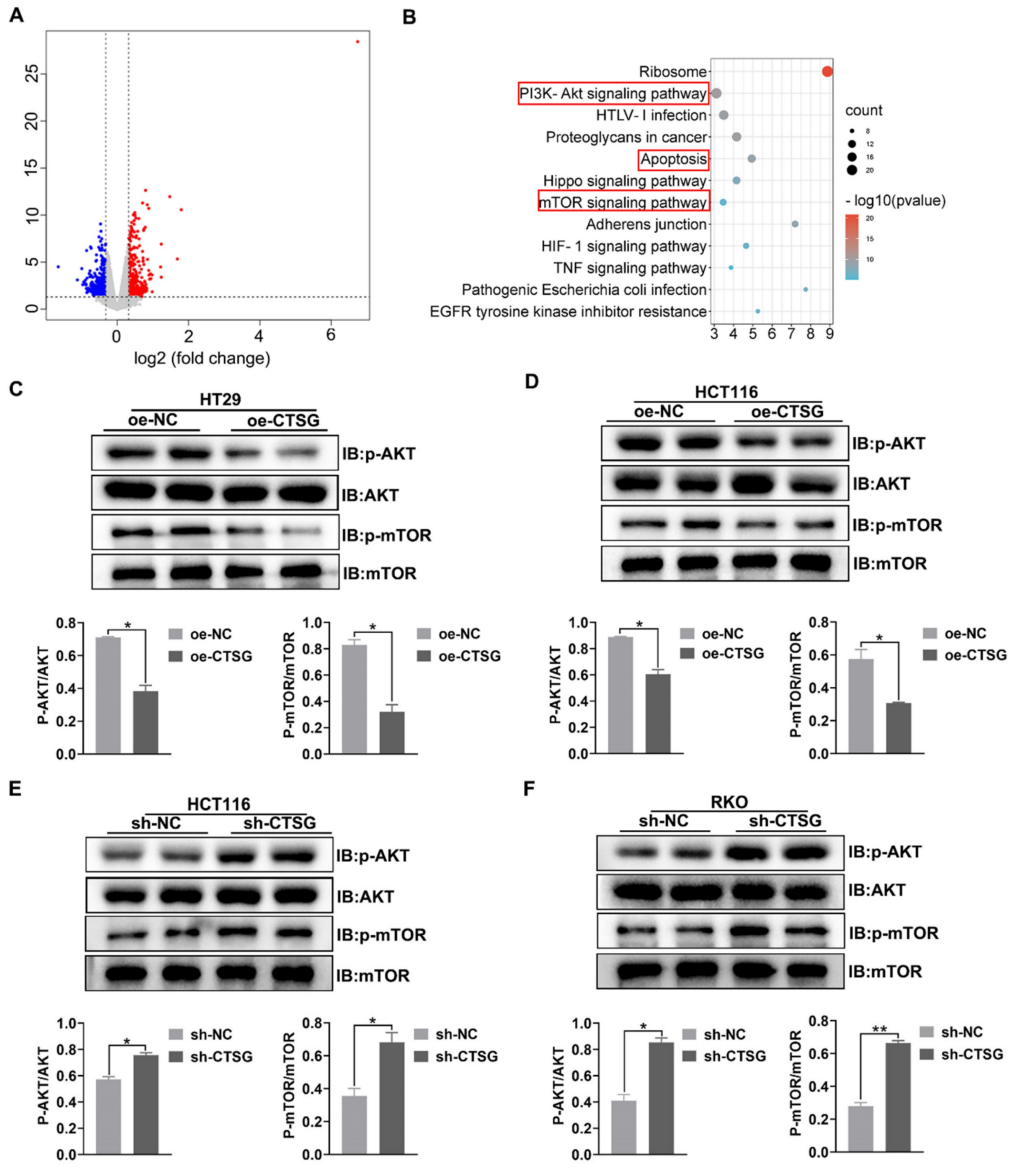
** CTSG mediates Akt/mTOR signaling pathway in CRC cell lines.** (A) Volcano plot of DEGs in control and stable CTSG overexpression HT29 cells by RNA sequencing; (B) KEGG pathway analysis enriched Akt and mTOR signaling pathways; (C**-**D) Western-blot and histogram analysis of p-Akt and p-mTOR level in CTSG stable over-expression HT29 (C) and HCT116 (D) cell lines. (E-F) Western-blot and histogram analysis of p-Akt and p-mTOR level in CTSG stable knockdown expression HCT116 (E) and RKO (F) cell lines. *P < 0.05, **P < 0.01.

To discover the potential mechanism that CTSG affects the growth of CRC cells. RNA-Seq was employed to profile the differentially expressed genes among the control and stable CTSG overexpression HT29 cells. The results showed that 789 differentially expressed genes (DEGs) were screened and displayed in the volcano plot **(Figure 5A) (Table S5)**. KEGG pathway analysis enriched PI3K/Akt, mTOR signaling mechanism, and apoptosis process (**Figure 5B**). To verify RNA-seq results, we performed western-blot assay to detect whether silencing or overexpression of CTSG would affect the expression of the related proteins. Western-blot analysis showed that CTSG overexpression might suppress the Akt phosphorylation. Moreover, mTOR phosphorylation levels, a downstream effector of PI3K/Akt signaling, were significantly reduced (**Figures 5C-D**). Nevertheless, CTSG silencing expression might increase Akt and mTOR phosphorylation (**Figures 5E-F**). The above data suggest that CTSG may be related to regulating CRC growth and apoptosis by modulating the PI3K/Akt signaling.

### CTSG inhibits the growth of CRC cells *in vivo*

**Figure 6:**
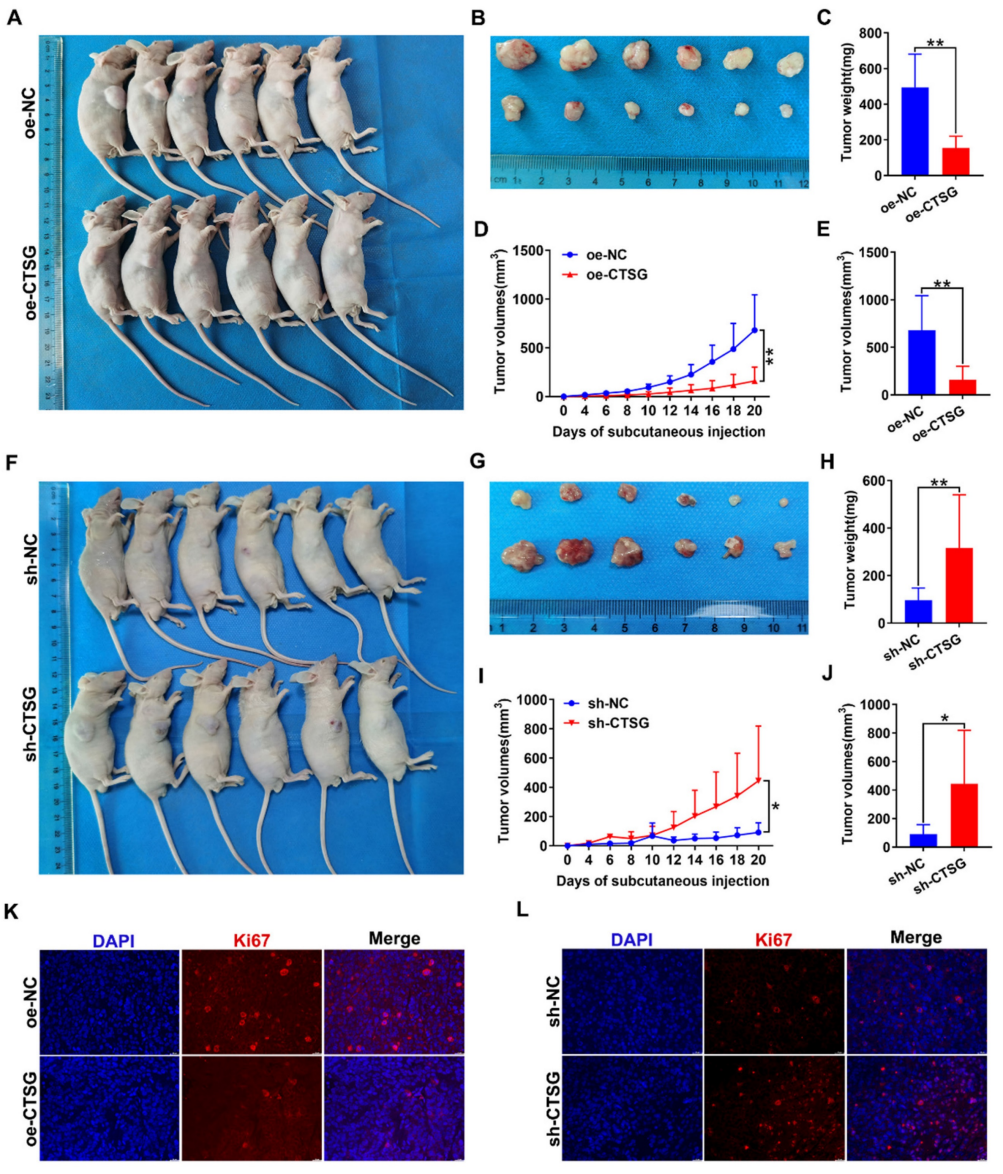
**CTSG inhibits CRC cells proliferation *in vivo*.** (A**-**E) CTSG overexpression inhibits proliferation: Photo of mice (A) injected with control and stable CTSG overexpression cells and tumor picture from mice (B), Tumor weight on the day the mice sacrificed (C), Tumor volume measured on every other day (D), and tumor volume on the day the mice sacrificed (E); (F**-**J) CTSG knockdown expression promotes proliferation: Photo of mice (F) injected with control and stable CTSG down-regulated expression cells and tumor picture from mice (G), Tumor weight on the day the mice sacrificed (H), Tumor volume measured on every other day (I), and tumor volume on the day the mice sacrificed (J); (K**-**L) Immunofluorescence staining for Ki67 in tumor tissues. Scale bar: 20 μm. *P < 0.05, **P < 0.01.

To discover whether CTSG influences the CRC growth cells* in vivo*, we conducted xenograft tumor development model. The CTSG stable overexpression HT29 cells or knockdown RKO cells and different control cells were subcutaneously administrated into mice to generate xenograft cancers. The results indicated that CTSG overexpression inhibits tumor growth (**Figures 6A-B**), and tumors with CTSG overexpression have lower weight (**Figure 6C**) and smaller sizes than the control group (**Figures 6D-E**). These results showed the opposite action in the control and CTSG knockdown group (**Figures 6F-J**). However, injection of CTSG stable silenced or overexpressed CRC cells had no substantial impact on body weight in nude mice (**Figures S4A-B**). The immunofluorescence staining confirmed that tumors with CTSG silencing expression have fewer Ki-67-positive cells percentage than control tumors (**Figure 6K**), and CTSG overexpression showed the opposite phenomenon (**Figure 6L**).

### MK2206 inhibits tumorigenesis caused by Akt/mTOR activation due to CTSG silencing *in vitro* and *in vivo*

**Figure 7:**
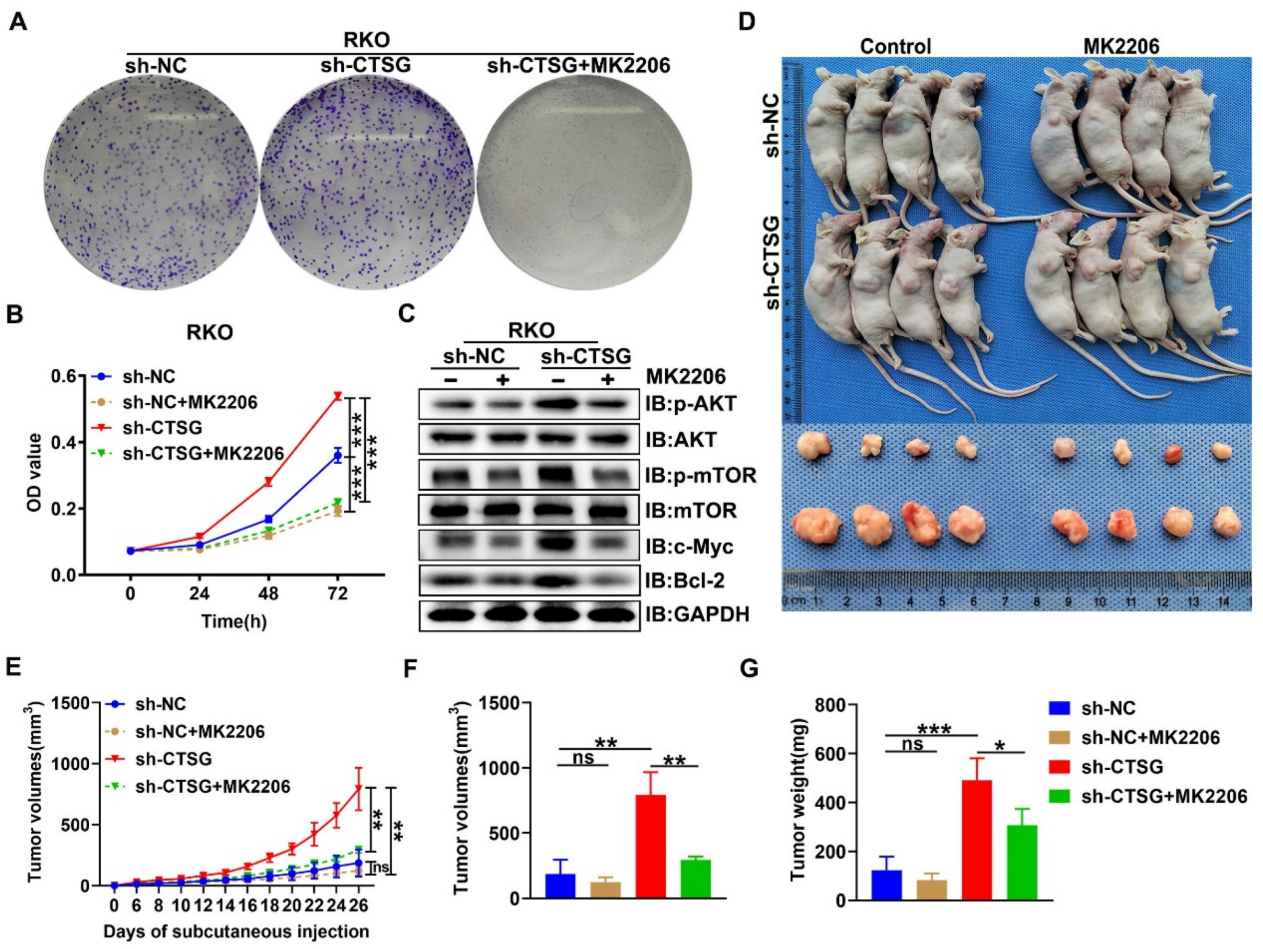
**MK2206 suppresses CRC cell proliferation induced by CTSG silencing *in vitro* and *in vivo*.** (A) Colony formation assay showed the effects of CTSG silencing cells treated with DMSO or MK2206 (5 μM) for ten days; (B) MTT assays showed the cell viability after treated with DMSO or MK2206 (5 μM); (C) The control cells and stable knockdown expression RKO cells were treated with DMSO or MK2206 (5 μM) for 24 h and then subjected to Western-blot analysis with the showed antibodies; (D**-**G) The control cells and stable knockdown expression RKO cells were injected subcutaneously into the flanks of mice, then treated with MK2206 (100 mg/kg) or equivalent volume of solvent. Mice and tumor pictures (D), tumor volume measured on every other day (E), tumor size (F), and weight (G) were recorded on the day the mice sacrificed. ns means no significant difference, *P < 0.05, **P < 0.01, ***P < 0.001.

Since Akt/mTOR signaling stimulation supports tumor development by regulating cell growth 33, we examined the functional consequence of CTSG absence-mediated activation of Akt/mTOR action. We next performed MTT and colony formation assay, and we observed that MK2206, a highly selective Akt suppressor 34, might obstruct the enhanced cell viability and colony formation capability caused by CTSG silencing expression (**Figures 7A-B**). Actually, we also demonstrated that MK2206 increased the apoptosis rate of CRC cell with CTSG knockdown expression (**Figures S5** and **Figures S6**). Western-blot analysis showed that down-regulated CTSG increased Akt and mTOR phosphorylation levels and promoted the c-Myc and Bcl2 protein expression, whereas MK2206 administration might reverse the promoting effect of silencing CTSG on AKT/mTOR signaling pathway (**Figure 7C**). Furthermore, we established a xenograft tumor growth model to investigate the MK2206 effect on CTSG deficiency on tumorigenesis *in vivo*. Our results demonstrated that MK2206 treatment significantly blocked CTSG knockdown-induced cell proliferation. As displayed in **Figures 7D-G**, tumors derived from mice injected with the stable knockdown expression RKO cells exhibited faster growth (**Figure 7E**) and higher weight (**Figure 7G**) than that injected with the control cells. These data reveal that CTSG absence exhibits cancer stimulative impacts through Akt/mTOR signaling pathway activation and Akt signaling pathway suppression by MK2206 abolishes CTSG silenced expression-induced up-regulation of Bcl2 and cell viability *in vitro* and *in vivo*. Moreover, SC79, an Akt activator, could promote cell proliferation and metastasis through positive regulation of Akt 35-37. Our results also showed that up-regulation of CTSG reversed SC79-induced CRC cell proliferation (**Figure S7**). In summary, these outcomes suggest that CTSG regulates CRC cell proliferation through the Akt/mTOR signaling mechanism regulation.

## Discussion

Along with the progress of chemotherapy and targeted medication, the clinical outcome of patients with CRC is substantially better than before, but it is unsatisfactory 11, 12. Although carcinoembryonic antigen (CEA) and other tumor markers have been widely used in CRC diagnosis and prognosis evaluation clinically, more efficient biomarkers are still needed to be identified for early diagnosis of CRC, and the study of therapeutic targets may be the main direction for further improvement of patient survival 1. CTSG belongs to the cathepsins gene family. It is one of the major components of neutrophil azurophilic granules and is involved in destroying intracellular and extracellular pathogens through nonoxidative pathways 38. It was a potential therapeutic target in various human diseases 39. Recently, studies focused on exploring the association between CTSG and malignant diseases 26-29, 40, 41. However, the CTSG role in CRC and its mechanism has not been well studied.

In the current investigation, we demonstrated that CTSG expression levels were markedly reduced in human CRC samples and several CRC cell lines. Early-stage CRC showed higher CTSG expression levels than the advanced stage, suggesting that CTSG is an inhibitor gene in CRC development and progression. Interestingly, we found that patients with increased CTSG expression had more favorable medical results than individuals with reduced CTSG expression and CTSG related to tumor size, location, and pathological stages in CRC. Consistent with the clinical significance, our data demonstrated that CTSG over-expression markedly suppressed viability and promoted apoptosis of CRC *in vitro* and *vivo*, tumor growth was induced, and apoptosis was suppressed after CTSG knockdown. It has been reported that CTSG is up-regulated in oral squamous cell carcinoma (OSCC), and CTSG over-expression might considerably suppress OSCC cell growth, migration, and invasion 29, 40. However, our results suggest that CTSG expression upregulation in CRC has no effect on CRC invasion and metastasis, and CTSG may not play the same role in different tissues. In addition, studies suggested that CTSG broad expression was observed in acute myeloid leukemia (AML), and CTSG might be an effective immunotherapeutic target for patients with AML 26, 41. In addition, CTSG has been informed as a cancer inhibitor gene in breast cancer 27 and bladder cancer 28. Our results also suggest CTSG would play as a tumor inhibitor gene in CRC, which is consistent with those studies.

In the current study, RNA-sequencing results showed that differentially expressed genes between the control and stable CTSG over-expression HT29 cell line enriched in PI3K/Akt, mTOR signaling mechanism, and apoptosis process. By transforming extracellular and environmental stimuli into intracellular signals that influence nutrient uptake, growth factors, metabolism, growth, apoptosis, angiogenesis, tissue development, and vesicle transport processes, the PI3K/Akt signaling pathway supports many cellular mechanisms 13, 14. It is also one of the important pathways related to CRC occurrence and progression, activation of this pathway correlated with cellular transformation, tumor progression, and drug resistance 15, 42. Our results confirmed that Akt and mTOR phosphorylation levels were substantially decreased after CTSG expression upregulation, while Akt and mTOR phosphorylation levels were raised after CTSG knockdown. Akt activation is also associated with resistance to chemotherapy 43. Targeting Akt may be an essential strategy for tumor prevention and therapy since abnormal Akt overexpression or activation has been shown in several malignancies, including ovarian, lung, and pancreatic cancer, and is related to higher cancer cell growth and survival 15. Therefore, reduced CTSG expression levels may be a factor in clinical chemotherapy resistance against CRC.

Apoptosis is the first kind of programmed cell death identified, and resistance to apoptosis is thought to be one of the fundamental characteristics of cancer 32. Previously, many studies have demonstrated that programmed cell death caused by apoptosis is a natural barrier to tumor progress 44-46. The Bcl2, one of the important cancer-associated markers, has a vital function in regulating apoptosis and survival; overexpression or abnormal activation of Bcl2 directly participates in maintaining various types of cancer cell growth and promoting therapeutic resistance 17, 18. Our study has indicated that Bcl2 expression was reduced, and apoptosis regulators, such as Caspase3 and Bax, were elevated after CTSG over-expression. However, after CTSG knockdown in CRC cells, these proteins expression levels were significantly increased. MK2206, an Akt inhibitor, has shown remarkable potential in treating hepatocellular carcinoma, testicular cancer, and breast cancer 47-49. Our results showed that CTSG down-regulated expression effects on tumor growth might be reversed, and Akt phosphorylation level and its downstream substrate mTOR decreased after using MK2206, indicating that CTSG may suppress CRC growth through Akt/mTOR signaling pathway. Previous studies have suggested that Bcl2 may act as Akt signaling downstream and is involved in tumor proliferation and apoptosis with Akt phosphorylation raising 19, 20, 50. Similarly, our results revealed that Bcl2 expression was reduced after using MK2206. These results demonstrated that CTSG promoted Bcl2-mediated apoptosis in CRC *via* Akt/mTOR signaling.

**Figure 8:**
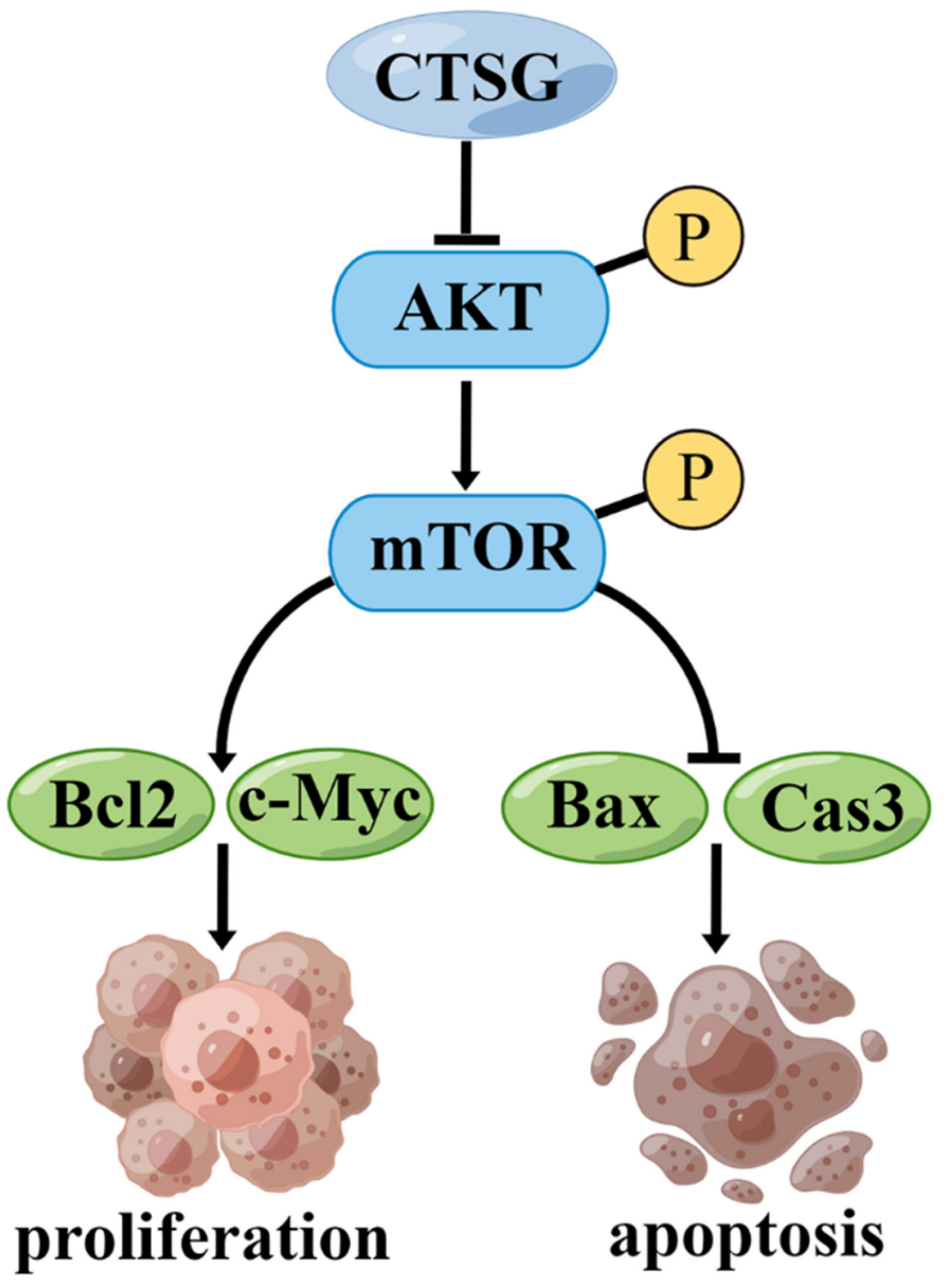
** A proposed mechanism that CTSG suppresses the proliferation and promotes apoptosis of CRC cells.** The figure is drawn by Figdraw (https://www.figdraw.com/).

However, this investigation has certain restrictions. First, more clinical samples should be collected to determine the association between CTSG expression and medical and pathological features in subjects with CRC. Second, we demonstrated that CTSG might inhibit CRC cell growth and promote apoptosis by regulating the Akt/mTOR/Bcl2 signaling mechanism **(Figure 8)**. However, the underlying mechanism by which CTSG regulates Akt activity remains unclear. Next, we will explore the potential mechanism by which CTSG regulates Akt/mTOR/Bcl2, particularly by constructing colorectal epithelial cell-specific CTSG-knockout mice.

## Conclusion

Our findings identify that CTSG has a tumor-inhibiting effect on CRC cells via controlling the activity of the Akt/mTOR/Bcl2 mediated anti-apoptotic signaling pathway. Consequently, these outcomes provide new insight that warrants future investigations into applying Akt inhibitors for precision therapy of patients with CRC and low CTSG expression.

## Supplementary Material

Supplementary figures and tables, table 5 legend.

Supplementary table 5.

## Abbreviations

CRC: Colorectal cancer
CTSG: Cathepsin G
Akt: Protein Kinase B
mTOR: mammalian target of rapamycin
TCGA: The Cancer Genome Atlas
IHC: Immunohistochemistry
KEGG: Kyoto Encyclopedia of Genes and Genomes
Bcl2: B-cell lymphoma 2
Bax: Bcl2-Associated X protein
OS: overall survival
SPF: Specific Pathogen Free
