# Supplementary material for: CTSG Suppresses Colorectal Cancer Progression through Negative Regulation of Akt/mTOR/Bcl2 Signaling Pathway
Source: Int J Biol Sci. 2023 Apr 17;19(7):2220–33. doi: 10.7150/ijbs.82000 (PMC10158020; doi:10.7150/ijbs.82000)
Supplement: Supplementary file 1 — Supplementary figures and tables, table 5 legend. [file ijbsv19p2220s1.pdf]

Supplementary Materials

Supplementary Figure 1.

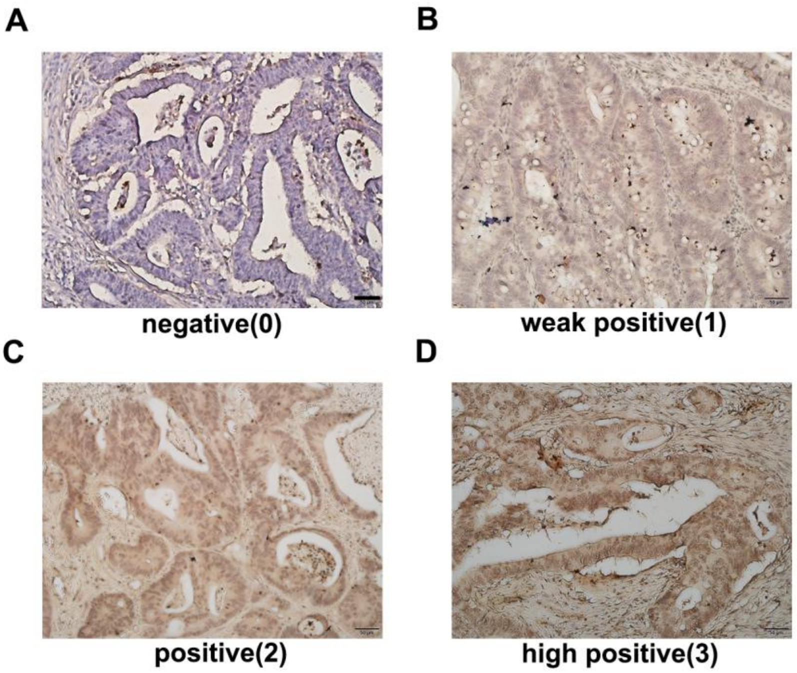

Figure S1. Four images represented IHC score for CTSG in 112 CRC tissues. (A-D) IHC score measured by ImageJ with Profiler plug-in (four intensity levels: 0-1 for low expression and 2-3 for high expression): negative(A), weak positive (B), positive (C), and high positive (D). Scale bar: 50μm.

Supplementary Figure 2.

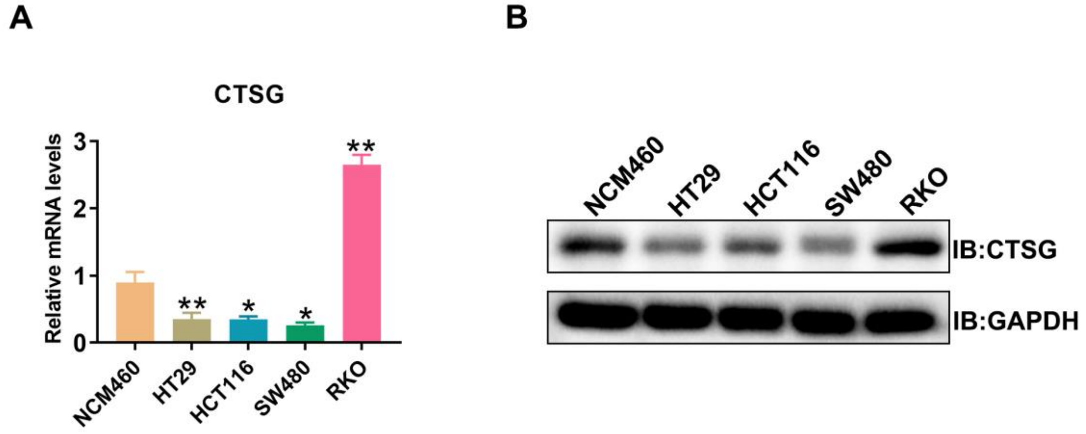

Figure S2. The endogenous CTSG expression in NCM460 and CRC cell lines. (A) The CTSG mRNA level in NCM460 and CRC cell lines. (B) The CTSG protein level in NCM460 and CRC cell lines. *P < 0.05, **P < 0.01.

Supplementary Figure 3.

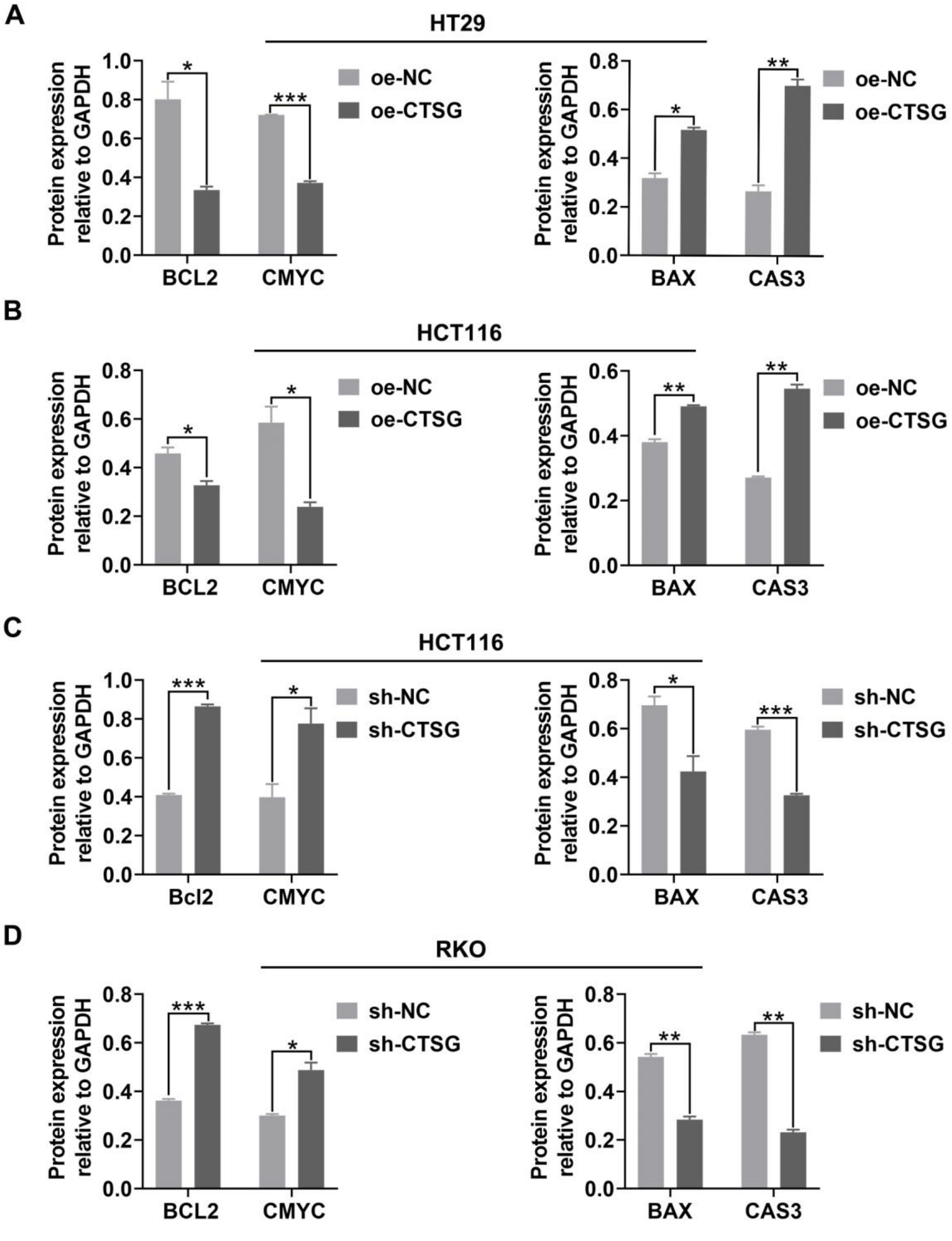

Figure S3. Histogram of quantitative analysis of bands. (A-B) Histogram analysis of protein level in CTSG stable over-expression HT29 (A) and HCT116 (B) cell lines. (C-D) Histogram analysis of protein level in CTSG stable knockdown expression HCT116 (C) and RKO (D) cell lines. **P* < 0.05, ***P* < 0.01, ****P* < 0.001.

Supplementary Figure 4.

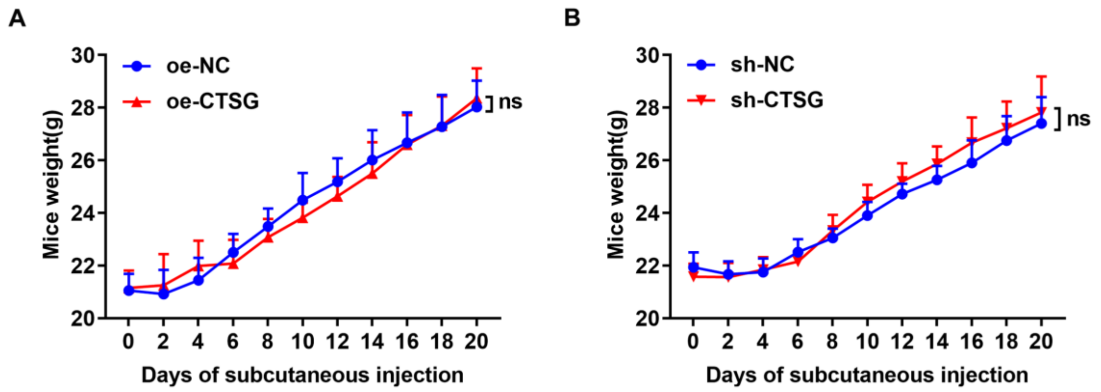

Figure S4. Mice weight in different treatment group. (A) Mice weight measured on every other day in control and overexpressed CTSG group. (B) Mice weight measured on every other day in control and down-expressed CTSG group. ns means no significant difference.

Supplementary Figure 5.

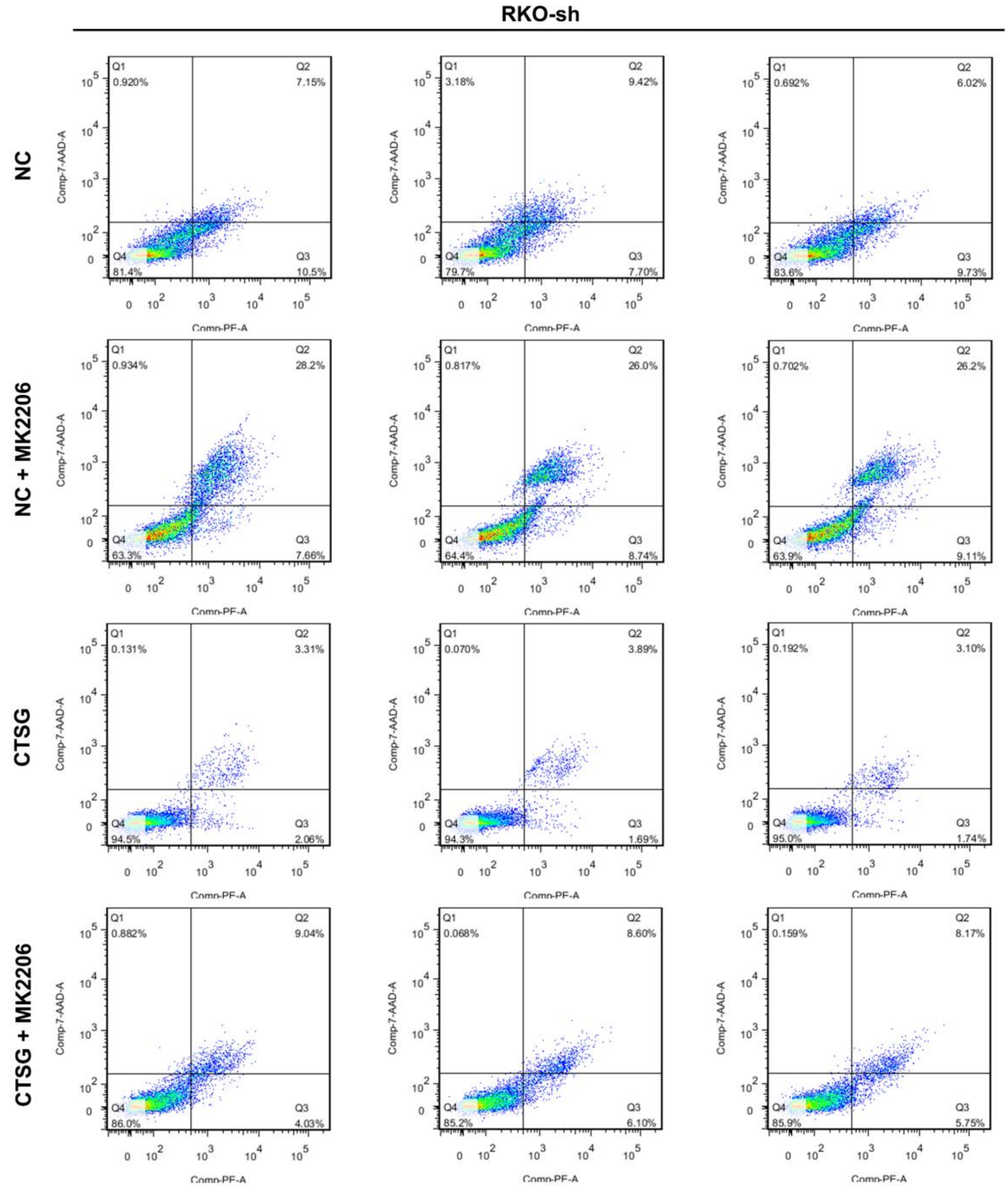

Figure S5. Flow cytometry assay indicates that MK2206 promotes cell apoptosis in CTSG stable knockdown expression RKO cells.

Supplementary Figure 6.

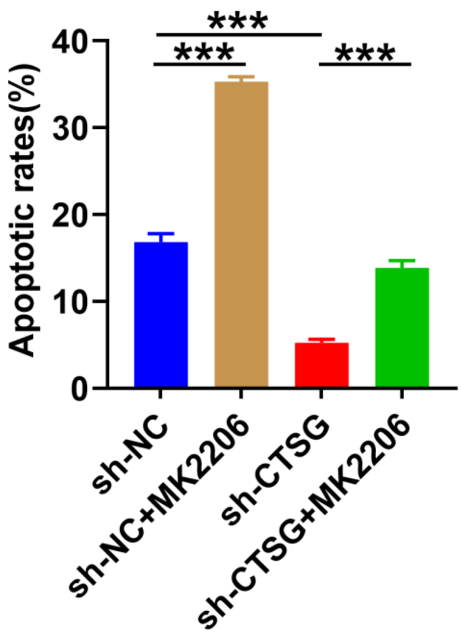

Figure S6. Histogram analysis of apoptosis rates of CTSG stable knockdown expression RKO cells with or without MK2206 treatment (5μM). ****P* < 0.001.

Supplementary Figure 7.

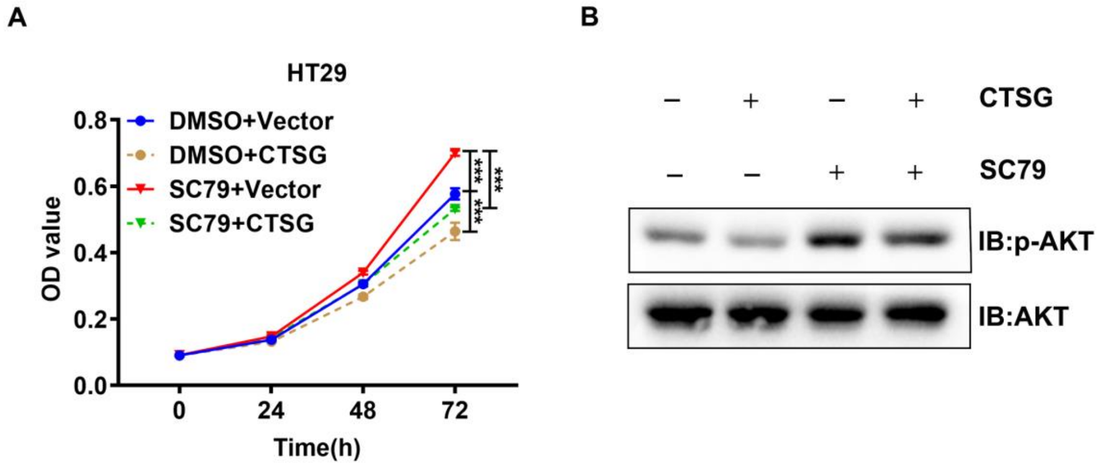

Figure S7. CTSG reverses SC79-induced CRC cell proliferation. A. MTT demonstrated that CTSG reversed the enhanced proliferative ability of HT29 cells after SC79 pretreatment for 2 hours (5 μM). B. CTSG reduced the increased AKT phosphorylation caused by SC79. ****P* < 0.001.

Table S1. The correlations between clinicopathological features and CTSG expression.

Variables	N (%)	CTSG high (%)	CTSG low (%)	*P*-value
Total Case	112	42 (37.5%)	70 (62.5%)	
Age (years)				
<65	67 (59.8%)	25 (22.3%)	42 (37.5%)	0.964
>=65	45 (40.2%)	17 (15.2%)	28 (25.0%)	
Sex				
Male	62 (55.4%)	24 (21.4%)	38 (33.9%)	0.768
Female	50 (44.6%)	18 (16.1%)	32 (28.6%)	
Tumor size				
<5cm	43 (38.4%)	28 (25.0%)	15 (13.4%)	
>=5cm	69 (61.6%)	14 (12.5%)	55 (49.3%)	<0.001
Location				
Left	47 (42.0%)	34 (30.4%)	13 (11.6%)	
Right	65 (58.0%)	8 (7.1%)	57 (50.9%)	<0.001
Stage				
I/II	57 (50.9%)	31 (27.7%)	26 (23.2%)	
III/IV	55 (49.1%)	11 (9.8%)	44 (39.3%)	<0.001

Table S2. Plasmids list.

Plasmid	Company
PCDH-Flag-GFP-CTSG	Youbio Biological Technology Co. LTD. China
PCDH-Flag-GFP	Youbio Biological Technology Co. LTD. China
pLKO-CTSG	Tsingke Biotechnology Co. LTD. China
pSPAX2	Shanghai GenePharma Co. LTD. China
pLP/VSVG	Shanghai GenePharma Co. LTD. China

Table S3. qPCR primers.

Gene	Forward primer (5' to 3')	Reverse primer (5' to 3')
36B4	ATCCCTGACGCACCGCCGTGA	TGCATCTGCTTGGAGCCCACGTT
CTSG	TGAGGCAGGGGAGATCATCG	TGGGTGTTTTCCCGTCTCTG

Table S4. The expression of CTSG in normal and tumor tissues.

	CTSG expression			
	Total	Low	High	*P*-value
Normal	41	13 (31.7%)	28 (68.3%)	<0.001
Tumor	112	70 (62.5%)	42 (37.5%)	

Table S5. A total of 789 DEGs screened from RNA-seq.

The data are in the excel named **Table S5**.
